# Supplementary material for: Qualitative evaluation of an edutainment intervention to prevent age-disparate transactional sex in Tanzania: Changes in educational aspirations and gender equitable attitudes towards work
Source: PLOS Glob Public Health. 2024 Apr 3;4(4):e0002527. doi: 10.1371/journal.pgph.0002527 (PMC10990206; doi:10.1371/journal.pgph.0002527)
Supplement: Attachment — Submitted filename: Response to Review.docx [file pgph.0002527.s001.docx]

Response to Review

Qualitative evaluation of an edutainment intervention to prevent age-disparate transactional sex in Tanzania: Changes in educational aspirations and gender equitable attitudes towards work

| **Reviewer Comment** | **Response** |
| --- | --- |
| 1. Thank you for the opportunity to review this interesting manuscript on the LINEA intervention. Some considerations for revisions are detailed below: | Thank you for taking the time to review our manuscript, we look forward to addressing your concerns. |
| 2. Minor comments: You use the abbreviation ADTS in the abstract only - please write this out in full. | In response to this comment we have replaced ADTS with age-disparate transactional sex throughout the abstract and made other cuts to remain within the word count. Pg. 2 lines 28-49 of the tracked changes version. |
| 3. Please briefly expand on the social science theory that informs edutainment in line 127. | Edutainment draws heavily on Albert Bandura’s (2004) Social Cognitive Theory which suggests that audience members are more likely to engage in new behaviours or modify existing behaviours in line with behaviours performed by characters if they like the characters or identify with them. We have added this and a reference on pg. 4 lines 129-132 of the tracked changes version. |
| 4. . On line 239 you refer to 20% transcripts - please add the actual number of transcripts dual coded. Were they coded in entirety or for particular themes? | We have updated this sentence to state that we dual coded 18 transcripts in their entirety. Pg. 6 line 46 of tracked changes version. |
| 5. Th authors indicate that of the 81 participants recruited, 59 (73%) partook in endline IDIs - what happened to the rest - lost to follow up, any reasons, e.g., relocation | The reasons for lost-to-follow-up were reported in full in our companion paper (Pichon et al. 2022). In response to this comment we have added a sentence summarising these reasons on pg. 7 lines 262-263 of tracked changes version: “Drop-out rates were highest among adult participants, the most common reasons being busy schedules and work-related travel.” |
| 6. Lines 250-260 can be simplified. | We have deleted some of the text describing study participants in response to this comment on pg. 7, lines 264-268 of tracked changes version. |
| 7. Major comments: 1. The manuscript comprises two broad categories - this requires some further refinement into a few more subthemes (subheadings) to improve the readability, flow and coherence. | Thank you for this helpful suggestion, we agree that subheadings improve readability, flow and coherence of the paper. Thus, we have added sub-headings to each section of the results.  In the section on long-term educational goals we have added the subheadings:   1. “Motivation to focus on studies” (pg. 8 line 282) 2. “Ability to move past life challenges” (pg. 8 line 307), and 3. “Avoiding sexual and reproductive health risks” (pg. 9 line 333)   In the second section on increased gender equitable attitudes about work we have added the sub-headings:   1. “Women working in traditionally male-dominated professions” (pg. 9 line 357), and 2. “Women’s motivation to engage in income generating activities” (pg. 10 line 399) |
| 8. Were there any negative perceptions or negative reported impacts of the intervention that were identified in transcripts? This feels like an important but missing piece especially as the RCT is rolled out. | Thank you for raising this important point. In response we have added a couple sentences to the limitations section on pg. 13 lines 498-502 which read: “We were particularly interested in exploring any potential negative consequences of the intervention and we did so through specific questions during the interview and by focusing on this aspect in our coding of the transcripts. No evidence of negative perceptions were identified and the limited negative reported impacts we found are thoroughly reported in the forthcoming process evaluation paper of the study.”  We have also added mention of this future paper on pg. 4 lines 149-150 of the tracked changes version. |
| 9. Were there any notable differences in perceptions across genders/age-groups? | We did analyse the data for differences between adolescent girls, women and men and this is included in the results. We hope that with the addition of sub-headings added in response to reviewer comment 7, and the descriptions of the sub-headings at the beginning of each section of the results this would be made clearer now.  In particular there were 3 areas where we analysed and reported by gender/age:  The first theme on long-term educational goals was specific to adolescent girls only and did not arise in transcripts with women and men caregivers. We have added this on pg. 7 lines 281-282 of the tracked changes manuscript.  The first part of the second theme on gender equitable attitudes towards work – women working in traditionally male-dominated professions – includes evidence from all 3 demographic groups and we did not find any notable differences between girls, women and men. We have added mention of this to pg. 10 lines 379-380 of the tracked changes version.  The final section of the second theme, however, arose solely in interviews with women. We have added this on pg. 9 lines 356-357 of the tracked changes version. |

**References**

Bandura, A. Social cognitive theory for personal and social change by enabling media. In: Singhal A, Cody MJ, Rogers EM, Sabido M, editors. Entertainment-education and social change. New York: Routledge; 2004. p. 75-96.

Pichon M, Carter DJ, Howard-Merrill L, Sono R, Gimunta V, Rutenge O, et al. A mixed-methods, exploratory, quasi-experimental evaluation of a radio drama intervention to prevent age-disparate transactional sex in Tanzania. Frontiers in Reproductive Health. 2022;4:1000853.
